# Intensive Insulin Therapy for Septic Patients: A Meta-Analysis of Randomized Controlled Trials

**DOI:** 10.1155/2014/698265

**Published:** 2014-06-18

**Authors:** Fang Song, Liu-Jun Zhong, Liang Han, Guo-Hao Xie, Cheng Xiao, Bing Zhao, Yao-Qin Hu, Shu-Yan Wang, Chao-Jin Qin, Yan Zhang, Deng-Ming Lai, Ping Cui, Xiang-Ming Fang

**Affiliations:** ^1^School of Medicine, Zhejiang University, Yuhangtang Road 866, Hangzhou 310058, China; ^2^Department of Anesthesiology, The First Affiliated Hospital, School of Medicine, Zhejiang University, Qingchun Road 79, Hangzhou 310003, China; ^3^Department of Surgical Oncology, The First Affiliated Hospital, School of Medicine, Zhejiang University, Qingchun Road 79, Hangzhou 310003, China; ^4^Department of Surgery, Children's Hospital, School of Medicine, Zhejiang University, Zhuganxiang 57, Hangzhou 310003, China

## Abstract

*Background*. Studies on the effect of intensive insulin therapy (IIT) in septic patients with hyperglycemia have given inconsistent results. The primary purpose of this meta-analysis was to evaluate whether it is effective in reducing mortality. *Methods*. We searched PubMed, Embase, the Cochrane Library, clinicaltrials.gov, and relevant reference lists up to September 2013 and including randomized controlled trials that compared IIT with conventional glucose management in septic patients. Study quality was assessed using the Cochrane Risk of Bias Tool. And our primary outcome measure was pooled in the random effects model. *Results*. We identified twelve randomized controlled trials involving 4100 patients. Meta-analysis showed that IIT did not reduce any of the outcomes: overall mortality (risk ratio [RR] = 0.98, 95% CI [0.85, 1.15], *P* = 0.84), 28-day mortality (RR = 0.66, 95% CI [0.40, 1.10], *P* = 0.11), 90-day mortality (RR = 1.10, 95% CI [0.97, 1.26], *P* = 0.13), ICU mortality (RR = 0.94, 95% CI [0.77, 1.14], *P* = 0.52), hospital mortality (RR = 0.98, 95% CI [0.86, 1.11], *P* = 0.71), severity of illness, and length of ICU stay. Conversely, the incidence of hypoglycemia was markedly higher in the IIT (RR = 2.93, 95% CI [1.69, 5.06], *P* = 0.0001). *Conclusions*. For patients with sepsis, IIT and conservative glucose management show similar efficacy, but ITT is associated with a higher incidence of hypoglycemia.

## 1. Introduction 

Sepsis has been a long withstanding issue in modern medicine that in too many instances leads to mortality. Every year in the United States there are approximately 750,000 documented cases, of which at least 225,000 are fatal [1]. Though there have been advances in intensive care, the mortality rate of patients with sepsis has remained between 20% and 30% over the past three decades [1, 2]. One pathophysiological component in septic patients is hypermetabolism, including perturbations of glucose metabolism resulting in hyperglycemia [3].

Hyperglycemia is prevalent in ICU patients, especially those with sepsis [4–6]. Hyperglycemia is associated with many adverse outcomes, including immune disorder, oxidative stress, susceptibility to infection, and endothelial dysfunction [7, 8]. Its impact is believed by research that has found hyperglycemia to be independently associated with increased mortality in patients with sepsis because it enhances the inflammatory response [6, 9]. Some randomized controlled clinical trials have attempted to determine whether intensive insulin therapy targeted on establishing normoglycemia could benefit septic patients [10–14].

In 2001, a randomized controlled trial showed that intensive treatment with insulin (80–110 mg/dL) resulted in a lower hospital mortality in the surgical ICU, which was attributed to a reduction of mortality in patients with sepsis [15]. Conversely, the VISEP study, the first to specifically investigate intensive insulin therapy for septic patients, found no significant reduction in mortality [10]. Then, further randomized controlled trials failed to replicate the mortality benefit in septic patients [16, 17]. Despite this continuing debate, the Surviving Sepsis Campaign included an upper limit for blood glucose of 180 mg/dL in their guidelines based upon systematic reviews of studies involving critically ill patients [18]. Although sepsis is the chief cause of death in ICUs, whether the impact and safety of intensive insulin therapy in septic patients are the same as those in critically ill patients is uncertain.

In order to clarify this matter, we conducted a meta-analysis to assess the use of intensive insulin therapy in managing glycemic control for septic patients. The primary purpose was to evaluate the effects of tight glycemic control on mortality stratified into four subgroups (90-day and 28-day mortality and hospital and ICU mortality).

## 2. Methods

### 2.1. Search Strategy

We searched for randomized controlled trials of intensive insulin therapy targeting euglycemia among septic patients in PubMed, Embase, the Cochrane Library, and clinicaltrials.gov dating up to September 2013 without language restriction. We used the exploded Medical Subject Heading (MeSH) terms “insulin,” “blood glucose,” and “hypoglycemic agents,” with the text words “hyperglycemia,” “insulin,” “blood glucose,” and “glycemic control,” for the intensive insulin filter. The MeSH term “sepsis” with the text words “sepsis,” “severe sepsis,” “septic shock,” and “septicemia” for the sepsis filter. Additionally, a highly sensitive search strategy described in the Cochrane Handbook was utilized for the randomized controlled trials filter [19]. The MeSH terms and text words were combined with the Boolean operator OR, and then the three filters were combined with the Boolean AND operator. We also checked the reference lists of retrieved reviews and clinical trials to identify additional studies.

### 2.2. Study Selection

Two investigators independently reviewed all of the titles and abstracts. Included articles met the following criteria: (1) trials had a randomized controlled clinical design, with or without blinding; (2) patients were adults with sepsis; (3) the intensive insulin therapy group had a targeted glucose concentration of ≤150 mg/dL, and the control group had a higher glucose level; and (4) the outcome measures included at least one of the following: mortality, severity of illness, length of ICU stay, and hypoglycemia (≤40 mg/dL). We also included data from trials in critically ill patients if the data concerning sepsis could be extracted.

### 2.3. Data Abstraction and Quality Assessment

Independently, the same two investigators abstracted the data and assessed the methodological quality of eligible trials. If there was any disagreement, a third investigator participated in a group discussion and made the final decision. The abstracted data was as follows: first author, year of publication, region or country, number of study site, sample size, population, patient age, history of diabetes mellitus, initial glucose level, targeted glucose level, and achieved mean glucose value. The primary outcome was mortality with a preference for 90-day mortality. If this was not reported in the outcome, we used hospital mortality, 28-day mortality, or ICU mortality, in that order. The secondary outcomes were severity of illness, length of ICU stay, and hypoglycemia. We also stratified mortality by 90-day and 28-day mortality, as well as hospital and ICU mortality in subgroup analyses.

The methodological quality was formally evaluated using the Cochrane risk of bias assessment tool, which incorporates random sequence generation, allocation concealment, blinding of participants and personnel, blinding of outcome assessment, incomplete outcome data, selective outcome reporting, and other potential sources of bias [19]. Each item was stratified into one of three categories: (1) high risk, which represented low quality, (2) low risk, which represented high quality, or (3) unclear, in which there was insufficient information to judge or the study did not involve this outcome.

### 2.4. Statistical Analysis

We used the Review Manager software to conduct the statistical analyses [20]. For each outcome measure, we used the relative risk (RR) for dichotomous data and the standardized mean difference (SMD) for continuous data. We used a random-effect model for all analyses which provides a more conservative pooled estimate than a fixed-effect model, considering the anticipated clinical heterogeneity among eligible articles [21]. Some data were presented with means and 95% confidence intervals, necessitating that we calculate standard deviations from the data provided. We assessed the heterogeneity among studies using Cochran's *Q*-test (*P* < 0.10 for statistical significance) and the *I* ^2^ statistic (*I* ^2^ value >50% for substantial heterogeneity). To eliminate the heterogeneity, we conducted either a sensitivity analysis or subgroup analysis.

## 3. Results

### 3.1. Literature Search

**Figure 1:**
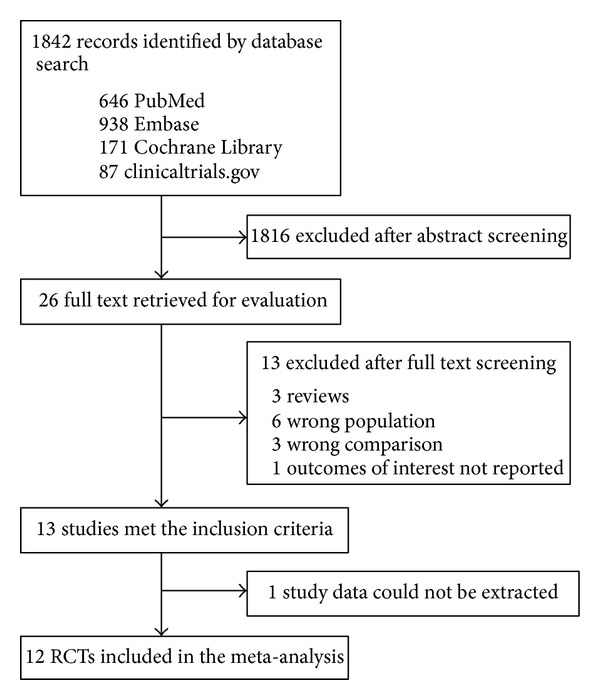
Flow diagram of study selection.

Our predefined search strategy yielded a total of 1,842 abstracts (Figure 1). After reviewing the titles and abstracts, we excluded 1,816 studies because they were nonrandomized controlled trials, not specific to septic patients or pertained to interventions other than intensive insulin therapy. We checked the full text of the remaining 26 articles. One trial that met our inclusion criteria was excluded because the data were presented in diagrams from which we were unable to abstract values [22]. No additional studies were found in the screened reference lists. Finally, 12 randomized controlled trials were included in our meta-analysis [10–14, 16, 17, 23–27]. One trial was only an abstract, despite contacting the authors to request a copy of the full article [23].

### 3.2. Study Characteristics

The 12 randomized controlled trials included 4,100 patients in all, of whom 2,094 were assigned to the intensive insulin group and 2,006 to the control group. The details of the included studies are listed in Table 1. For the intervention, all the trials used tight glycemic control (80–110 mg/dL) except for one trial used that used two glycemic subcategories: 80–110 mg/dL or 120–150 mg/dL [23]. Because our inclusion criterion was an intensive insulin therapy group targeting a glucose concentration of ≤150 mg/dL, we combined the data of the two glycemic subcategories for mortality analysis. The mean glucose concentration of included patients varied significantly among eligible trials, from 130 mg/dL to 216 mg/dL. The proportion of patients with septic shock ranged widely (32.7–100%). In 3 trials, the baseline parameters of the septic patients were undocumented in the intensive and the control groups [17, 23, 24]. Most of the included trials used a specific method of random sequence generation (Table 2). None of the trials met the “blinding of participants and personnel” bias criterion and thus were rated as high-risk. On the contrary, all studies were identified as low risk because they did not have “incomplete outcome data.”

### 3.3. Primary Outcome: Mortality

**Figure 2:**
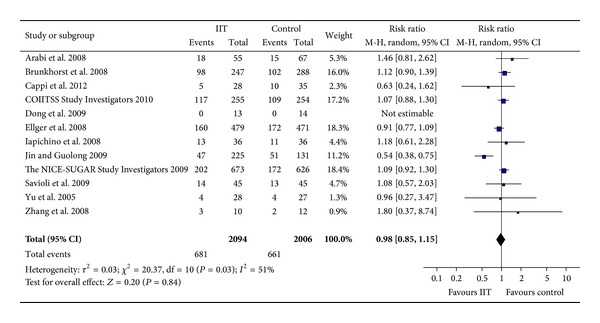
Forest plot with all-cause mortality showing no significant difference between IIT and control group (RR = 0.98, 95% CI [0.85, 1.15]). IIT: intensive insulin therapy. CI: confidence interval. M-H: Mantel-Haenszel.

The all-cause mortality was reported in the 12 randomized controlled trials [10–14, 16, 17, 23–27]. There were 681/2,094 (32.5%) deaths in the intensive insulin intervention group and 661/2,006 (33%) in the control group. Meta-analysis showed that the rate of death did not differ significantly between the two groups (RR = 0.98, 95% CI [0.85, 1.15], *P* = 0.84) (Figure 2).

**Figure 3:**
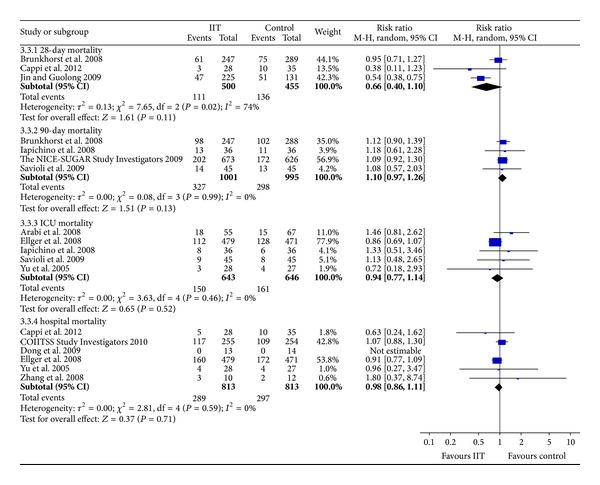
Meta-analysis showing no significant difference between IIT and control groups, with mortality being stratified into 28-day, 90-day, ICU, and hospital mortality. IIT: intensive insulin therapy. CI: confidence interval. M-H: Mantel-Haenszel.

In the subgroup analysis, there was no significant difference in 28-day mortality (RR = 0.66, 95% CI [0.40, 1.10], *P* = 0.11), 90-day mortality (RR = 1.10, 95% CI [0.97, 1.26], *P* = 0.13), ICU mortality (RR = 0.94, 95% CI [0.77, 1.14], *P* = 0.52), or hospital mortality (RR = 0.98, 95% CI [0.86, 1.11], *P* = 0.71) (Figure 3).

The statistical heterogeneity was substantial for all-cause mortality (*I* ^2^ = 51%; *P* = 0.03) and for 28-day mortality (*I* ^2^ = 74%; *P* = 0.02). Because we could not acquire the full text of the study by Jin and Guolong [23], we did not have sufficient information to evaluate its methodological quality. Therefore, we excluded this trial and the heterogeneity for all-cause mortality was resolved (*I* ^2^ = 0%; *P* = 0.51). The heterogeneity was still significant for 28-day mortality. We noted that the Cappi trial had a wide confidence interval due to its small sample size [14]. Thus, we identified this trial as an outlier in the 28-day mortality. Even with this adjustment, the results of all-cause mortality (RR = 1.05, 95% CI [0.96, 1.14], *P* = 0.33) and 28-day mortality (RR = 0.95, 95% CI [0.71, 1.27], *P* = 0.74) did not change significantly.

### 3.4. Secondary Outcomes: Severity of Illness, Length of ICU Stay, and Hypoglycemic Events

**Figure 4:**
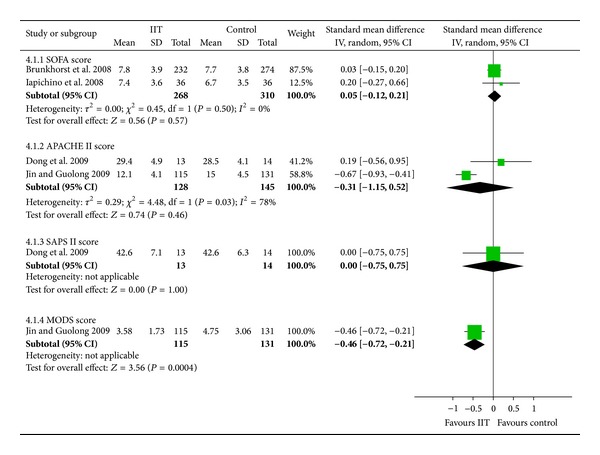
Forest plot with severity of illness showing no significant difference between IIT and control groups in SOFA, APACHE II, and SAPS II scores, with a reduction of MODS score with IIT. IIT: intensive insulin therapy. SD: standard deviation. CI: confidence interval. IV: inverse variance.

The included trials used the SOFA (Sequential Organ Failure Assessment) score, APACHE II (Acute Physiology and Chronic Health Evaluation II) score, SAPS II (Simplified Acute Physiology Score II), and MODS (Multiple Organ Dysfunction Score) to evaluate severity of illness after intensive insulin therapy. Five trials reported SOFA score [10–13, 16], two studies reported APACHE II score, and only the MODS [23] and SAPS II [27] scores were reported in only one study each. The data about SOFA score could only be extracted from two trials that included 578 participants [10, 11]. The pooled estimate in the intensive insulin group was similar to that in the control group (SMD = 0.05, 95% CI [−0.12, 0.21], *P* = 0.57) with no statistical heterogeneity (*I* ^2^ = 0%; *P* = 1.00) (Figure 4). Similarly, there was no significant difference in APACHE II score (*P* = 0.46) and SAPS II (*P* = 1.00). The MODS was lower in the intensive insulin group, but its methodological quality was low. This suggests that intensive insulin therapy does not reduce the severity of sepsis.

Five trials reported the length of ICU stay as an outcome [10, 11, 16, 23, 26]. It should be noted that in four of the trials the data were presented as the median (interquartile range) [10, 11, 16, 26], each with no statistically significant difference between the intensive insulin and control groups. The remaining trial with unclear methodological quality found that intensive insulin therapy resulted in a shorter ICU stay, but the result was dubious [23]. We did not merge the medians (interquartile range), because we could not determine whether they had a normal distribution [19].

**Figure 5:**
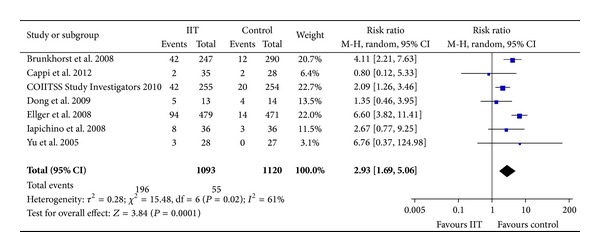
Forest plot showing that IIT increased the risk of hypoglycemia. IIT: intensive insulin therapy. CI: confidence interval. M-H: Mantel-Haenszel.

For the occurrence of hypoglycemia, we extracted data from seven trials including 2,213 participants [10, 11, 13, 14, 16, 25, 27] (Figure 5). There were 196/1,093 (17.9%) hypoglycemic events in the intensive insulin group and 55/1,120 (4.9%) in the control group. The intensive insulin group had a higher rate of hypoglycemia than the control group (RR = 2.93, 95% CI [1.69, 5.06], *P* = 0.0001), and there was substantial heterogeneity across the trials (*I* ^2^ = 61%, *P* = 0.02). We identified the Ellger et al. trial as an outlier [25], and its exclusion resolved the heterogeneity (*I* ^2^ = 19%, *P* = 0.29) but did not change the pooled estimate (RR = 2.44, 95% CI [1.59, 3.72], *P* < 0.0001).

### 3.5. Sensitivity Analysis

Through performing several sensitivity analyses (the trials with “low risk” of “random sequence generation” and “allocation concealment”, the trials with >500 patients, the trials with septic shock data, and studies with excluding unclear baseline characteristics) [17, 23, 24], the overall effect size was found to change minimally regardless of mortality or hypoglycemia (Table 3).

### 3.6. Publication Bias

We were unable to evaluate the publication bias, in part, due to the appearance of heterogeneity and also due to the small number of the included trials in each comparison.

## 4. Discussion 

We conducted a meta-analysis of randomized controlled trials of intensive insulin therapy for septic patients, which showed no reduction of mortality overall nor in any of the subgroups (28-day and 90-day mortality; ICU and hospital mortality). Likewise, in terms of the severity of illness, the intensive insulin group did not show a statistically significant difference from the control. Though one trial found that the intensive insulin therapy group had a shorter ICU stay than the control group, its methodological quality was vague. Due to the data format, we could not merge the other studies with the length of ICU stay. Intensive insulin therapy, however, notably increased the episodes of hypoglycemia. We found substantial heterogeneity in the pooled analysis of mortality and hypoglycemia, but the results remained the same when the heterogeneity was removed using sensitivity analysis.

During the past 30 years, no effective new therapies appeared, despite that our understanding of the pathophysiologic features of sepsis has advanced [28]. A number of observational studies indicate that hyperglycemia is associated with a higher mortality rate, in particular, sepsis-induced hyperglycemia rather than mortality due to preexisting diabetes mellitus [6]. The landmark randomized controlled trial showed that intensive insulin therapy (targeting 80–110 mg/dL) reduced hospital mortality in septic patients [15], so clinicians enthusiastically received it as an effective therapy for these patients. Unfortunately, subsequent randomized controlled trials failed to confirm this beneficial effect.

Several prior reviews investigated the effect of intensive insulin therapy (IIT) for a general population of ICU patients [29–31]. Though we obtained similar results regarding the effect of IIT on mortality and risk of hypoglycemia, there are some differences in our findings compared with the prior reviews. The previous reviews concentrated on a general population of ICU patients. Solyemez Wiener et al. [29], Kansagara et al. [30], and Griesdale et al.'s [31] trials were grouped by type of ICU (medical ICU, surgical ICU, and mixed ICU). Kansagara et al.'s [30] trials were also grouped by type of patients (myocardial infarction and stroke); however, we focused on patients with sepsis. Even though septic patients are intermixed with other ICU patients, they possess distinct treatment and prognostic and clinical outcomes as compared with other ICU patients. Therefore, the effect of intensive insulin therapy for ICU patients is not necessarily congruent with the effect for septic patients. In addition, we classified the outcome of mortality into four subgroups (90-day, 28-day, hospital, and ICU mortality) and added the outcome of severity of illness and the length of ICU stay that were not evaluated in the prior reviews.

We included one specific study [25] that contained a database of two randomized controlled trials [15, 32], which used identical protocols and were implemented at the same center but one year apart. Thus, we treated these studies as a consecutive study. This study [25] showed that intensive insulin therapy reduced the ICU mortality in septic patients who stayed in the ICU for at least three days, but there was no statistically significant difference in septic patients who stayed for less than three days. They performed this subgroup analysis to specifically consider patients whose intensive care was limited or who were withdrawn from intensive care within 3 days of admission to the ICU. Another trial, the Brunkhorst et al. trial [10], showed no effect of intensive insulin on mortality in septic patients who stayed in the ICU at least three or five days. The remaining trials did not stratify by the length of stay in the ICU. Taking the authentic clinical practice environment into consideration, we pooled the overall ICU stay and found no significant difference.

Perhaps the failure to find a benefit with intensive insulin therapy can be attributed to several factors. It remains unclear whether hyperglycemia is a cause of increased mortality or is just a marker of an increased risk of death; it may even be a normal response [6]. Finfer's [33] latest review states that “Until quite recently stress hyperglycemia was seen as a normal and possibly beneficial physiological response to promote cellular glucose uptake” (pp: 1–6).

In agreement with previous studies, our meta-analysis demonstrated that intensive insulin therapy carries a markedly increased risk of hypoglycemia. Hypoglycemia has been reported to have an independent association with increased mortality in patients with sepsis [34, 35]. Considering the increased risk of death due to increased hypoglycemia and the findings that intensive insulin therapy does not reduce mortality, the use of IIT as a strategy to maintain normoglycemia remains unclear.

Septic patients are apt to suffer from a striking increase in blood glucose variability, which causes endothelial dysfunction, oxidative stress, and organ dysfunction [6, 9, 36, 37]. Though the mean blood glucose concentration is similar among trials, the degree of glucose variability may be quite different. Several observational studies have shown that blood glucose variability is independently associated with an increased mortality rate, even more than continuous hyperglycemia [9, 35]. The glycemic lability index, which is calculated from continuous glucose monitoring, reveals the inherent variability better than the standard deviation of the mean blood glucose value [35]. The included trials mainly used sampling at a predefined time, rather than monitoring 24-hour continuous glucose levels, resulting in insufficient data to determine the relationship between glucose variability and mortality rate.

There are several limitations to our meta-analysis. Septic patients who survive to hospital discharge still have a high risk of death in the following months and years, which has been suggested in many studies [38]. The NICE SUGAR trial found that intensive insulin therapy increased mortality compared with the control group at 90 days, but not at 28 days [17]. Furthermore, the VISEP study, which found that intensive insulin therapy did not reduce mortality in septic patients, was discontinued early due to an excess risk for hypoglycemia [10]. Trials included in our meta-analysis mainly contained data gathered before hospital discharge and follow-up was limited. Therefore, our results may not be appropriate for long-term prognosis.

The glucose target range of the most trials in this meta-analysis was 80–110 mg/dL. Perhaps, a result favoring intensive insulin therapy could have been found to occur if a higher concentration range was used in the intensive insulin group, in comparison with uncontrolled hyperglycemia. Restricted by the lack of adequate data, we were not able to stratify trials based on the type of ICU and the proportion of calories provided parenterally or to evaluate secondary outcome measures, such as severity of illness, length of ICU stay, and cost. Also, there is the possibility of publication bias in our review.

Unfortunately, none of the included randomized controlled trials used blinding of participants and personnel. Several trials had few mortality events, so we could not detect small differences between groups. Furthermore, the patient characteristics, blood glucose control, and coexisting interventions varied across the included studies.

## 5. Conclusion 

Overall, the results of our meta-analysis suggest that intensive insulin therapy provides no benefit for septic patients. The highly sensitive search strategy, searching multiple databases and clinicaltrials.gov, searching publications written in any language, and performing subgroup analyses and sensitivity analyses, provides strength and rigor for our meta-analysis. Future reviews of septic patients may require individual patient data and more data that captures outcome measures.

## Figures and Tables

**Table 1:** Characteristics of included randomized controlled trials.

First author, year (country)	Number of study sites	Sample size	Population, %	Mean age, (years)	Diabetic, %	Initial glucose, mg/dL	Glucose goal, mg/dL	Glucose achieved, mean (SD), mg/dL
Cappi 2012 (Brazil) [14]	1	63	Severe sepsis Septic shock	53	24	IIT 144 [97–182]^∗^ Control 141 [101–160]^∗^	IIT 80–110 Control 140–180	IIT 99 (18) Control 155 (39.6)

COIITSS 2010 (France) [16]	11	509	Septic shock 100	64	NA	IIT 216 (NA) Control 204 (NA)	IIT 80–110 Control 180–200	IIT 120–140 Control 140–160

Savioli 2009 (Italy) [12]	3	90	Severe sepsis 65.6 Septic shock 34.4	61	13.3	IIT 175 (101) Control 160 (74)	IIT 80–110 Control 180–200	IIT 112 (23) Control 159 (31)

Iapichino 2008 (Italy) [11]	3	72	Severe sepsis 19.4 Septic shock 80.6	62.3	17	IIT 137 (45) Control 151.7 (36.6)	IIT 80–110 Control 180–200	IIT 110 (17) Control 163 (29)

Brunkhorst 2008 (Germany) [10]	18	537	Septic shock 100	64.6	30	IIT 130 [108–167]^∗^ Control 138 [111–184]^∗^	IIT 80–110 Control 180–200	IIT 112 (NA) Control 151 (NA)

Ellger 2008 (Belgium) [25]	1	950	Severe sepsis 51.4 Septic shock 48.6	62	14	IIT 163 (73) Control 161 (70)	IIT 80–110 Control 180–200	IIT 106 (26) Control 150 (30)

Yu 2005 (China) [13]	1	55	Sepsis	46	NA	IIT 153 (61) Control 151 (65)	IIT 80–110 Control 180–200	IIT 103 (22) Control 198 (29)

Dong 2009 (China) [27]	1	27	Septic shock 100	44	0	IIT157 (45) Control 159 (39.6)	IIT 74–110 Control 112–150	IIT 108 (27) Control 148 (34)

NICE-SUGAR 2009 (Australia and New Zealand—Canada) [17]	42	1299	Severe sepsis Septic shock	60.2	20.1	IIT 146 (52.3) Control 144 (49.1)	IIT 81–108 Control 180 or less	IIT 115 (18) Control 144 (23)

Arabi 2008 (Saudi Arabia) [24]	1	122	Severe sepsis Septic shock	52.4	40.0	IIT 195 (75) Control 211 (81)	IIT 80–110 Control 180–200	IIT 115 (18) Control 171 (34)

Zhang 2008 (China) [26]	1	22	Sepsis	66.3	27.5	IIT 165 (55) Control 200 (100)	IIT 80–110 Control 130–150	IIT 119 (7.6) Control 141 (7.9)

Jin 2009^#^ (China) [23]	14	356	Severe sepsis Septic shock	65.7	NA	IIT NA Control NA	IIT 80–110; 120–150 Control 180–200	IIT 80–110: 99 (31); 120–150: 133 (34) Control 189 (40)

IIT: intensive insulin therapy; NA: not available from article or author.

*Median (interquartile range).

^#^Abstract only.

**Table 2:** Risk of bias in included randomized controlled trials.

Study	Random sequence generation	Allocation concealment	Blinding of participants and personnel	Blinding of outcome assessment	Incomplete outcome data	Selective reporting	Other sources of bias
Cappi 2012 [14]	Low risk	Low risk	High risk	Low risk	Low risk	Low risk	Low risk
COIITSS 2010 (France) [16]	Low risk	Low risk	High risk	Low risk	Low risk	Low risk	High risk^a^
Savioli 2009 [12]	Low risk	Unclear	High risk	Low risk	Low risk	Low risk	Low risk
Iapichino 2008 [11]	Low risk	Unclear	High risk	High risk	Low risk	Unclear	Low risk
Brunkhorst 2008 [10]	Low risk	Low risk	High risk	High risk	Low risk	Low risk	High risk^a,b^
Ellger 2008 [25]	Low risk	Low risk	High risk	Low risk	Low risk	Low risk	Low risk
Yu 2005 [13]	Unclear	Unclear	High risk	Low risk	Low risk	High risk	Low risk
Dong 2009 [27]	Unclear	Unclear	High risk	High risk	Low risk	Unclear	Low risk
NICE-SUGAR 2009 (Australia and New Zealand—Canada) [17]	Low risk	Low risk	High risk	Low risk	Low risk	Low risk	High risk^c^
Arabi 2008 [24]	Low risk	Low risk	High risk	Low risk	Low risk	Low risk	High risk^d^
Zhang 2008 [26]	Unclear	Unclear	High risk	Low risk	Low risk	Unclear	Low risk
Jin 2009^∗^ [23]	NA	NA	NA	NA	NA	NA	NA

*Abstract only.

NA: not available from article or author.

^a^The two treatment groups differ in the use of medications other than insulin.

^b^Intensive insulin therapy was terminated early because of increasing hypoglycemic events.

^c^Inclusion used subjective criteria.

^d^It had a different baseline.

**Table 3:** Sensitivity analysis.

Outcome	Number of studies	Number of patients	RR (95% CI)	*P* value
Low risk of random sequence generation and allocation concealment				
Mortality	6	3478	1.04 (0.95–1.15)	0.40
Hypoglycemia	4	2059	3.27 (1.62–6.57)	0.0009
Trials containing >500 patients				
Mortality	4	3293	1.04 (0.94–1.14)	0.44
Hypoglycemia	3	1996	3.81 (1.89–7.69)	0.0002
Patients with similar baseline				
Mortality	9	1910	1.00 (0.89–1.13)	0.97
Hypoglycemia	7	2213	2.93 (1.69–5.06)	0.0001
Patients with septic shock				
Mortality	4	1533	1.03 (0.89–1.18)	0.69
Hypoglycemia	4	1535	2.97 (1.72–5.12)	<0.0001

RR: risk ratio; CI: confidence interval.
